# Priority in in the Use of Salol

**Published:** 1889-04

**Authors:** Richard H. Day

**Affiliations:** Baton Rouge, La.


### PRIORITY IN IN THE USE OF SALOL.

### German vs. American, "Progress of Medicine;" an Unjust Discrimination. Why is it?

LETTET FROM PROF. RICHARD H. DAY, M. D., NEW ORLEANS POLYCLINIC.

*Editor Daniel's Texas Medical Journal:*

The following qoutation is from the Philadephia "Medical Register" of March 23, 1889:

"The latest information from abroad is to the effect that Mr. Lowenthal has discovered a new remedy for cholera in Salol, his paper having been presented to the Academy by Prof. Cornil, presenting the results of investigations of Mr. Lowenthal in this matter. His experiments had been confined to animals, and, of course, the action of the remedy upon man cannot be positively stated at this date.

"The value of Salol in several intestinal affections has been demonstrated, and if in addition to this, we have in it a remedy for the scourge of cholora, it will be twice welcome." etc.

I enclose to you with this letter, a copy of reprint of my short article on the use of salol in the treatment of typhoid fever, which was published in the *Journal of the American Medical Association.*

The date of the use of salol in the treatment of typhoid fever by myself, clearly shows, that I was among the first, *if not the first*, to use it in that disease; and such was its prompt, decided and phenomenal success, that I deemed it my duty, to place at once before the medical profession of this country, my experience of its use—and yet although publishedin the journal of the association—and many of the reprints distributed to many of my confreres, and sent to several medical journals, in order that the remedy might be fully tried by the profession—and in localities, in our country where typhoid fever is so prevalent and destructive—all of our medical journals so far as I know, have failed to make any comment upon my article, or to ask the profession to consider and test the results of my experience.

Had my experience emanated in *Germany* or *France*, or any other *foreign country*—and been heralded by the name of some Professor, I trow, that our medical journals would not have been slow in publishing the information—and making it known to all of our reading physicians.

Mr. Editor, can you tell how, or, why it is that the labors and work and experience of our country physicians are thus ignored by our journals?

Why not encourage and stimulate their labors in the field of medicine, that their good works may be known and recognized; and they, if meritorious, receive the distinctions and the honors accorded to metropolitan professors and physicians?

The great mass of physicians all over this wide country, must be educated, stimulated, and worked up to the high standard of the present requirements of medicine, if we would make our profession in this country as in Europe, useful, respected and influential. This I conceive to be the duty of our journals—what say you?

Fraternally,

RICHARD H. DAY, M. D.

Baton Rouge, La., March, 1889.

